# Profylo: A Python Package for Phylogenetic Profile Comparison and Analysis

**DOI:** 10.1007/s00239-025-10280-6

**Published:** 2025-10-29

**Authors:** Martin Schoenstein, Pauline Mermillod, Arnaud Kress, Odile Lecompte, Yannis Nevers

**Affiliations:** 1 Complex Systems and Translational Bioinformatics (CSTB), Department of Computer Science, UMR7357 ICube, University of Strasbourg, CNRS, Centre de Recherche en Biomédecine de Strasbourg, Rue Eugène Boeckel 1, 67000 Strasbourg, France; 2 Bioinformatics & Genomics Est Platform (BiGEst-ICube)UMR 7357Centre de Recherche en Biomédecine de Strasbourg, ICube, University of Strasbourg, CNRS, Rue Eugène Boeckel 1, 67000 Strasbourg, France

**Keywords:** Comparative genomics, Phylogenetic profiling, Coevolution, Python package

## Abstract

Phylogenetic profiling, involving the analysis of presence-absence of orthologs in a set of species, is a way to infer functional association between genes through co-evolutionary patterns. Since its inception, numerous methods have been described to construct phylogenetic profiles, evaluate their similarity, or identify clusters of co-evolving genes. However, few of these methods are available as downloadable software. We present Profylo, a phylogenetic profiling toolkit made available as an open-source Python 3.0 package. Profylo implements seven methods for comparing phylogenetic profiling, four algorithms for identification of co-evolving clusters, as well as tools to help with their analysis, including visualization features. We take advantage of the variety of methods implemented in Profylo to benchmark their ability to predict functional relationships between human genes, using different datasets. Finally, we demonstrate the utility of the package with an example case study of the presence-absence of all protein-coding genes in the human genome. Profylo is available on GitHub at https://github.com/MartinSchoenstein/Profylo.

**Supplementary Information:**

The online version contains supplementary material available at 10.1007/s00239-025-10280-6.

## Introduction

Phylogenetic profiles are vectors representing presence or absence of orthologs of a gene in a list of species. Introduced by (Pellegrini et al. 1999), phylogenetic profiling refers to methods that use similarity between profiles to identify genes involved in the same biological pathway or that are functionally associated. The underlying assumption is that such genes will tend to follow similar evolutionary trajectories. Based on this assumption, phylogenetic profiling analyses assume that genes that co-evolve, and share similar phylogenetic profiles, are more likely to interact together and to be involved in the same biological pathway or process. Phylogenetic profiles can then be used to search for genes that have co-evolved with genes of interest (Tabach et al. 2013), or with a trait with a given presence-absence profile (Nevers et al. 2017). They can also be used to isolate modules of co-evolving genes (Dey et al. 2015)—groups of genes that have similar profiles. These analyses can be used to gain knowledge about the structural complex or biological process in which one or more genes are involved through the guilty-by-association principle.

Over the years, phylogenetic profiles have been successfully applied to the functional annotation of prokaryotic genomes (Zheng, Roberts, and Kasif 2002) and the identification of new components of molecular pathways (Zheng, Roberts, and Kasif 2002; Ramazzina et al. 2006). In eukaryotes, they have been used to identify new molecular players in the eukaryotic cilium (Avidor-Reiss et al. 2004; Hodges et al. 2011; Nevers et al. 2017), mitochondrial complexes (Li et al. 2014), the kinetochore complex (van Hooff et al. 2017), and small RNA pathway cofactors (Tabach et al. 2013). Similar approaches have also been used to identify genes linked to traits such as left–right asymmetry (Szenker-Ravi et al. 2022) or diet (Hecker et al. 2019).

A key methodological challenge of phylogenetic profiling lies in the way profiles are compared. Essentially, they are binary profiles (1 if an ortholog exists in a species, 0 otherwise). Accordingly, earlier works presenting these methods relied on basic methods of binary vector comparison, such as the Hamming similarity (Pellegrini et al. 1999) or the Jaccard distance (Nevers et al. 2017; Glazko and Mushegian 2004). A common criticism of these similarity measures, however, is that they are blind to species relatedness and sampling bias when certain clades are over-represented (Cokus et al. 2007). Therefore, other approaches used more elaborate measures of similarity: the Pearson correlation coefficient (that can also measure anticorrelation and works on continuous vectors) (Glazko and Mushegian 2004; Tabach et al. 2013), Mutual Information (Date and Marcotte 2003) and dimensionality reductions like Singular Value Decomposition (SVD) (Franceschini et al. 2016). The benefit of the latter is the possibility to account for redundancy of information carried by close species. Finally, some approaches have been designed to account for the phylogenetic relationships between species, such as the Phylogenetic Co-occurrence Score (PCS) (Dey et al. 2015) and the co-transition score (Dembech et al. 2023). Both methods use a species tree to order the vector, then create a new vector that represents transitions in presence-absence on the ordered one. This vector represents individual evolutionary events of gene loss or gain, which are compared under these similarity measures.

As a result of these continuous developments made over two decades, the number of potential tools for analysis of phylogenetic profiling is now quite large. However, not all of the methods are made available as public code, making it difficult for users to apply them to their own datasets. Furthermore, although most of the newly developed methods have been shown to perform well on their own benchmarks, the absence of easy-to-access implementations has prevented an exhaustive comparison of methods. Moreover, results of phylogenetic profiling depend on the target clade and the scope and quality of the genomes that are considered (Deutekom et al. 2022). A more accessible implementation of phylogenetic profiling comparison methods would facilitate comparisons between methods and across different sets of parameters, to better evaluate the factors affecting the performance of different methods.

Beyond the question of the similarity metrics, there is a general lack of consistent strategy to extract biologically relevant data from a phylogenetic profiling analysis. Two approaches can be used. The target approach starts from a given profile or gene, and looks for genes with a similar profile. In this case, the use of the p-value as implemented by (Dembech et al. 2023) in the co-transition score can help identify meaningful matches. Another approach is the identification of groups of genes with strong similarities through clustering methods. Clusters extracted in this way can be visualized through binary heatmaps (as seen in (Dey et al. 2015)) or matrices of filled dots (Tran et al. 2018), sometimes with an accessory species tree that can help infer eventual gain–loss events. Some dedicated visualization tools, like Matreex (Rossier et al. 2023), draw on phylogenetic profile frameworks to provide a compelling visualization of gene evolution, but are not specifically designed for phylogenetic profiles. Overall, few methods provide an out-of-the-box way to extract and visualize profiles, as they are often made *ad hoc* for specific publications.

To address these issues, we developed Profylo, a Python library and command-line tool dedicated to the processing, comparison and analysis of phylogenetic profiles. Profylo implements many of the previously described algorithms for profile comparisons, providing a way to retrieve profiles similar to a target and to extract co-evolutionary modules. It also includes visualization tools to explore the results. Here, we give a brief description of the software, provide benchmarks of implemented metrics to assess their ability to retrieve known functional association between genes, and describe an example use case of the library.

## Methods

### Library Implementation

Profylo is written in Python 3. It relies on the following libraries: biopython (1.84) (Cock et al. 2009), numpy (2.1.3) (Harris et al. 2020), pandas (2.2.3) (McKinney 2010), scikit-learn (1.5.2) (Pedregosa et al. 2011), scipy (1.15.2) (Virtanen et al. 2020), networkx (3.4.2) (Hagberg, Swart, and Schult 2008), matplotlib (3.9.4) (Hunter 2007), seaborn (0.13.2) (Waskom 2021), urllib3 (2.2.3) urllib3 · PyPI, goatools (1.4.12) (Klopfenstein et al. 2018), ete3 (3.1.3) (Huerta-Cepas et al. 2016) and markov-clustering (0.0.6.dev0).

The version numbers given here correspond to the ones used in the analysis presented in this work.

#### Phylogenetic Profile Distance or Similarity Metrics

We implemented the following methods as measures of similarity or distance between phylogenetic profiles:

**Jaccard distance.** A measure of distance between binary profiles, computed as follows:

$$J\left( {X,Y} \right) = 1 - \frac{{\left| {X_{1} \cap Y_{1} } \right|}}{{\left| {X_{1} \cup Y_{1} } \right|}}$$

where ${X}_{1}$ and ${Y}_{1}$ is the set of species with a presence in profiles X and Y.

We used Scipy’s Jaccard implementation.

**Hamming distance.** A measure of distance between binary profiles that takes into account unshared presence/absence, computed as follows:

$$H\left( {X,Y} \right) \, = \,\frac{{c\left( {0,1} \right) + c\left( {1,0} \right)}}{n}$$

where *c(i,j)* is the number of occurrences of *X[k]* = *i* and *Y[k]* =*j*, and *n* the length of profiles.

We used Scipy’s Hamming implementation.

**Pearson correlation.** An index reflecting a linear relationship between two profiles:

$$P\left( {X,Y} \right) = \frac{{\mathop \sum \nolimits_{i = 1}^{n} \left( {X_{i} - \overline{X} } \right)\left( {Y_{i} - \overline{Y} } \right) }}{{\sqrt {\mathop \sum \nolimits_{i = 1}^{n} \left( {X_{i} - \overline{X} } \right)^{2} } \sqrt {\mathop \sum \nolimits_{i = 1}^{n} \left( {Y_{i} - \overline{Y} } \right)^{2} } }}$$

We used Scipy’s Pearson correlation implementation.

**Mutual information.** A measure of the degree of probabilistic dependence between two variables, here two binary profiles:

$$I\left( {X,Y} \right) = \mathop \sum \limits_{i = 0}^{1} \mathop \sum \limits_{j = 0}^{1} \frac{{|X_{i} \cap Y_{j} |}}{N}\log \frac{{|X_{i} \cap Y_{j} |}}{{|X_{i} |\left| {Y_{j} } \right|}}$$

where ${X}_{i}$ and ${Y}_{j}$ are the sets of species with value i and j in profiles X and Y.

**SVD-Phy.** A measure of distance between profiles as introduced in (Franceschini et al. 2016). The phylogenetic profile matrix M is first decomposed using Singular Value Decomposition (SVD) yielding the matrix factorization M = USV’. This decomposition is performed using the SVD implementation in the numpy library. We use the option *full_matrices* = *False* so that when performing SVD on n*m matrix the size of the resulting U matrix is also n × m, as in the original publication. The resulting matrix is then truncated to retain the first C column, defined as the percentage of *m*. Then, we compute the Euclidean distance between each pair of rows of the matrix U, each row representing the profile of a gene.

For the benchmark presented in the manuscript, we chose the default value of 0.5.

**PCS.** A measure of similarity between phylogenetic profiles based on shared transition as introduced in (Dey et al. 2015). Vectors representing the presence-absence transitions in a tree-ordered profile are used. Given a binary transition vectors T1:(0 1), T2:(1 0), T3:(00 11) and T4:(11 00):

$${\text{PCS}}\left( {{\text{X}},{\text{Y}}} \right)\, = \, \mathop \sum \limits_{i = 1}^{n} ( T1_{{X_{i} }} = T1_{{Y_{i} }} ) + \mathop \sum \limits_{i = 1}^{n} \left( {T2_{{X_{i} }} = T2_{{Y_{i} }} } \right) + \mathop \sum \limits_{i = 1}^{n} \left( {T3_{{X_{i} }} = T3_{{Y_{i} }} } \right)w + \mathop \sum \limits_{i = 1}^{n} \left( {T4_{{X_{i} }} = T4_{{Y_{i} }} } \right)w - p [\mathop \sum \limits_{i = 1}^{n} \left( {T1_{{X_{i} }} \ne T1_{{Y_{i} }} } \right) + \mathop \sum \limits_{i = 1}^{n} \left( {T2_{{X_{i} }} \ne T2_{{Y_{i} }} } \right) + \mathop \sum \limits_{i = 1}^{n} \left( {T3_{{X_{i} }} \ne T3_{{Y_{i} }} } \right)w + \mathop \sum \limits_{i = 1}^{n} \left( {T4_{{X_{i} }} \ne T4_{{Y_{i} }} } \right)w]$$

where *w* is a specific weight given to transition supported by two species on each side and *p* a specific weight to control the effect of unshared transitions.

**Co-transition score.** A measure of similarity between phylogenetic profiles based on shared transition as introduced in (Dembech et al. 2023). Vectors representing the presence-absence transitions in a tree-ordered profile are used:

$$COTR\left( {X,Y} \right) \, = \,\frac{c - d}{{T_{x} - T_{y} + \left| {c - d} \right|}}$$

With *c* the number of concordant transitions and *d* the number of discordant transitions. *Tx* and *Ty* are the number of transitions in each vector.

#### Graph Analysis and Clustering

Profylo implements multiple ways to obtain phylogenetic profile clusters.

##### Graph Based Approaches

In the graph based approaches, genes are represented by nodes and the similarities between profiles are represented as edges between nodes. All graph processing is implemented using the networkx Python library. Three methods of graph clustering are implemented:

###### Connected Component

Only similarity between profiles over a threshold value are retained as edges. Then, connected components in the resulting graphs are identified with networkx and those with cardinality more than 2 are returned.

###### Label Propagation

Only similarity between profiles over or under a threshold value are retained as edges. Then, the graph is input to an unweighted label propagation algorithm. Nodes sharing the same labels are returned as clusters.

###### Markov Clustering

This strategy uses a fully connected graph, whereby edges are weighted by their similarity value. The graph is then input to markov clustering, using the implementation in the mcl Python library.

##### Hierarchical Clustering

Hierarchical clustering is performed using a distance matrix as obtained using the methods described above, with the implementation in the scipy library. Users can either obtain the full dendrogram, specify a number of clusters they want, or alternatively use a specific distance value to obtain an optimal number of clusters.

#### Species Tree Processing

Species trees were processed through the BioPhylo from the BioPython (Cock et al. 2009) and the ete3 libraries (Huerta-Cepas et al. 2016).

For a given profile, a species tree annotated with the ancestral state in Dollo parsimony framework is constructed as follows:

For generation of an annotated species tree corresponding to a module, leaves are annotated by phylogenetic profile state: 0 or 1. Values are propagated through the tree, with a 1 if at least one descendant node has a value of 1, or 0 instead. This is done through a recursive algorithm that stops at the node corresponding to the last common ancestor. The number of loss events implied by such a tree, designed here as the parsimony score, is calculated during the process by counting the number of losses.

For a complete module, this process is done for all profiles in a module and the average value for each node is retained.

#### Graphical Outputs

Graphs are generated via custom functions built using the seaborn Python library.

### Data Acquisition

All phylogenetic profiles used in this work were obtained from the 2023 release of OrthoInspector 3.5 (Nevers et al. 2019), using *Homo sapiens* as the query species and all species in the Eukaryota and Transverse databases as targets. To constitute the EukModel dataset, only eukaryotic species of the Transverse database were retained. Corresponding species trees were obtained from the OrthoInspector database in the Newick format, through API calls to the OrthoInspector website. They were originally obtained from the NCBI Taxonomy database in May 2023.

Pairs of human inparalogs were obtained from the OrthoInspector Eukaryota database by extracting all proteins that formed an inparalog group relative to any other species in the database. This list was used to exclude the pairs from analyses in a subset of the benchmarking experiments and in the example application.

### Benchmarking

KEGG Pathway data were downloaded from the KEGG website on 01/14/2025 (Minoru Kanehisa et al. 2025; M. Kanehisa and Goto 2000; Minoru Kanehisa 2019).

Benchmarking was carried out considering only the human proteins represented in the KEGG Pathway entries. Pairs of proteins sharing a pathway were considered as positive, and protein pairs not sharing a pathway as negative.

The performance of each method was measured as follows. First, from a similarity or distance matrix obtained from any of the implemented methods, we ordered all pairs of proteins according to their similarity, so that most similar profiles had the highest rank and the most dissimilar pair the lowest rank. Then, at each position in this ranked list, we computed the number of positive pairs found at this rank or above and the precision of all pairs at this rank or above. For benchmarks including all pairs we considered the first 10,000 pairs, for benchmarks excluding paralogs we considered the first 2000 pairs. Area under the curve (AUC) was computed on these data using the auc function of the scikit-learn package.

For benchmarking Profylo’s parsimony score, we first computed this score for every human gene, as in the species tree processing section. Then, for each pair, we selected the minimal score of genes and compared distributions of positive and negative pairs (as described above). Differences between distributions (H1:positive pairs have higher scores than negative) was tested with the one-sided Mann–Whitney U-test, as implemented in the scipy package.

### Case Study of the Human Phylogenetic Profiles

The case study was performed on the phylogenetic profiles of human genes extracted from the Eukaryota database. Pairwise similarities between profiles were computed using Profylo’s distance_profiles function with the “PCS” method with confidence parameter of 1 and penalty parameter of 0.3. Modules were obtained using label propagation in a graph where the edges represented similarities between genes higher than a threshold of 7 through the make_modules function of Profylo. Edges between paralogs were removed from the graph before generating the modules with the exclude_pairs parameter.

Statistics about the modules were computed using the Profylo descriptive functions (phylogenetic_statistics, go_enrichment). Gene Ontology term (Ashburner et al. 2000) enrichment was done using the go_enrichment function based on goatools (Klopfenstein et al. 2018), with Gene Ontology terms from the Gene Ontology resource (Gene Ontology Consortium et al. 2023). The five GO terms related to Molecular Function, Cellular Component, and Biological Process associated with the lowest corrected p-values were extracted.

The functional analysis of the clusters was supplemented by a study of the enrichment in modules and pathways from the KEGG resource. A p-value was calculated for each module and pathway using a hypergeometric test and corrected using the Bonferroni method. Only corrected p-values below 0.05 were considered.

Annotated gene trees were visualized with the beta version of the latest release of phylo.io (Robinson et al. 2016) at https://beta.phylo.io.

## Results

### Software Overview

**Fig. 1:**
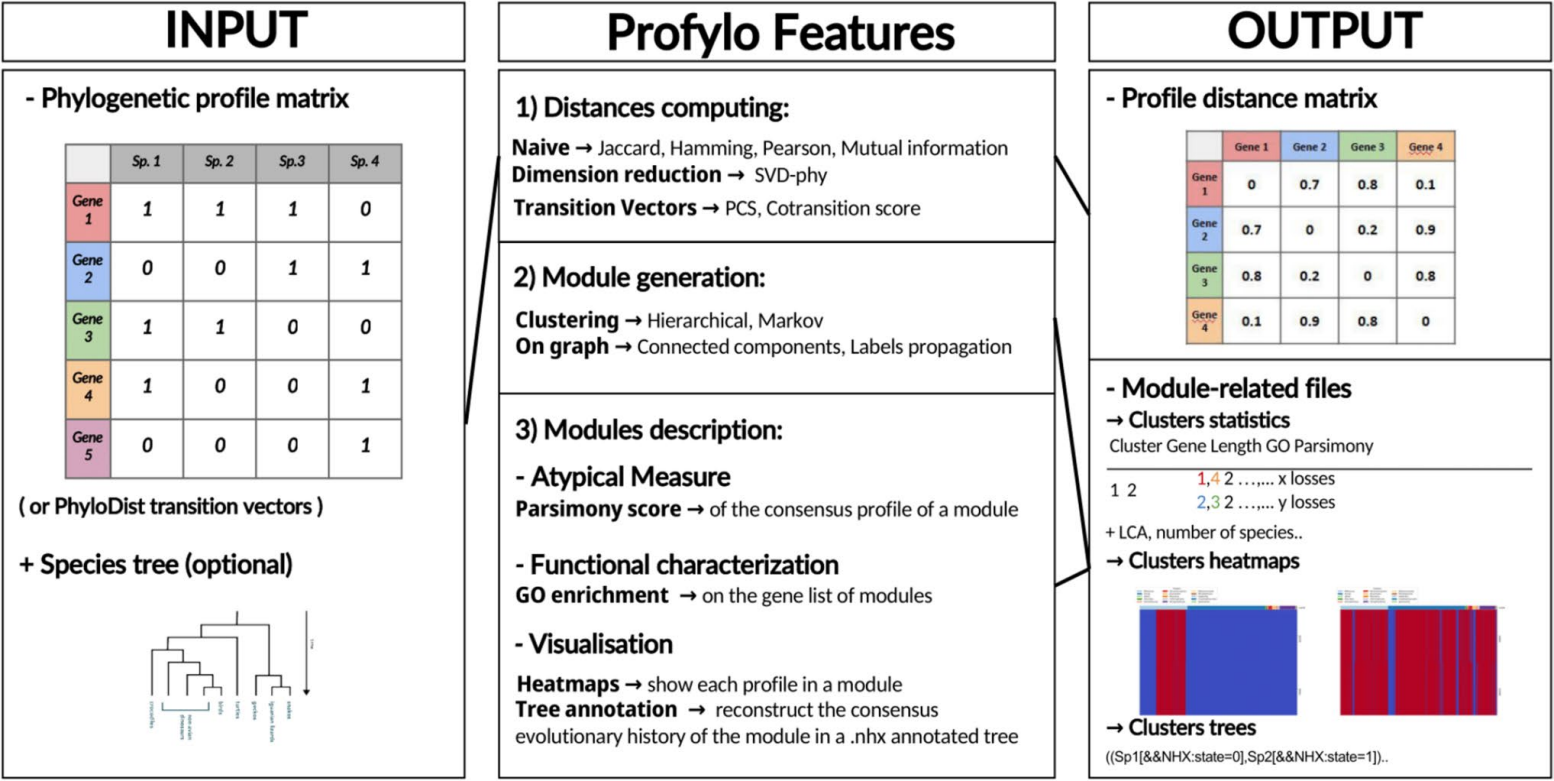
Profylo overview. Profylo takes as input a phylogenetic profile matrix and optionally, a species tree. Its features cover three steps of a phylogenetic profile analysis workflow. 1. Distance or similarity computation, which generates a similarity or distance matrix. 2. Module generation, which implements an algorithm for identifying groups of similar profiles. 3. Module description: descriptive and graphical representations of the identified modules

Profylo is available as an open source Python package, distributed through a GitHub repository (https://github.com/MartinSchoenstein/Profylo). It can be used through a command-line interface, from which users can obtain distance matrices and clustering results on their own phylogenetic profiles. However, it is mainly intended to be used through its Python API, which offers a wider variety of tunable parameters. Profylo takes phylogenetic profiles as input (Fig. 1), in a comma or tab-separated file format with genes as rows, and organisms as columns. The profiles themselves can be discrete (simple presence-absence) with a value of 1 or 0, or continuous where the value is then a measure of local sequence similarity.

The user can also provide a species tree in Newick format. This optional input is necessary for the taxonomy-based similarity metrics, for parsimony computation and some of the data exploration features.

#### Similarity Measures

The main objective of Profylo is to calculate several similarity metrics for comparing phylogenetic profiles from the literature available to the community in a single software package. In total, it implements seven distance and/or similarity metrics described in the literature. These measures are described in Table 1.

**Table 1:** Description of the seven metrics implemented in Profylo

Measure	Input type	Output (*ranges from most to least similar)	Reference
Jaccard	Discrete profiles	Matrix in range [0,1]	(Jaccard 1901)
Hamming	Discrete profiles	Matrix in range [0,1]	(Pellegrini et al. 1999)
Pearson correlation	Discrete or continuous profiles	Matrix in range [1,-1]	(Pearson 1895; Glazko and Mushegian 2004; Tabach et al. 2013)
Mutual Information	Discrete profiles	Matrix in range [1,0]	(Shannon 1948; Date and Marcotte 2003)
SVD-Phy	Discrete or continuous profiles	Matrix in range [0, + ∞]	(Franceschini et al. 2016)
PCS	Discrete profiles Species tree	Matrix in range [+ ∞,-∞]	(Dey et al. 2015)
cotr-score	Discrete profiles. Species tree	Matrix in range [1,-1] P-value matrix	(Dembech et al. 2023)

Using these measures, Profylo can compute the all-against-all similarity matrix for the phylogenetic profiles given as input. As an alternative, Profylo can also compute a reduced version of the whole similarity matrix, focused on a set of user-provided target genes (some-against-all) which may be used to identify genes having co-evolved with specific target genes without comparing all profiles to each other, thus reducing the volume of computation. This is designed for users interested in finding genes that are functionally associated to their target and not in a global analysis at the proteome level. This option can also be leveraged to identify genes that have co-evolved with a phenotype with a known presence-absence distribution—using the profile of that phenotype rather than that of a gene.

#### Downstream Module Identification

Once a phylogenetic profile similarity matrix is obtained, it is not always straightforward to identify groups of genes that have co-evolved together, especially in a hypothesis-free framework. Profylo integrates several strategies to isolate modules of genes sharing high similarity from the distance matrix.

The graph_module function is based on a simple graph analysis strategy. It first builds a graph from the distance matrix, where genes of the same species are the nodes, and the edges represent their profile similarity, considering only those over a specified threshold. Two strategies are implemented to extract modules from such a graph: a connected components identification or the use of a label propagation algorithm (see methods).

Profylo also implements two unsupervised clustering approaches to automatically extract modules without having to specify a threshold beforehand: a Markov clustering algorithm based on a graph representation and hierarchical clustering directly based on the distance matrix.

To facilitate user analysis, Profylo can output some descriptors for each module: a consensus profile, the average number of species with orthologs and the Last Common Ancestor’s clade, based on a hypothesis of vertical descent. Profylo also computes a measure of the parsimony of the consensus profile for each module, which we call the parsimony score. This score is equal to the number of events (losses) needed to explain the profile in a maximum Dollo parsimony framework. The informativeness of this score for identifying functionally associated genes is benchmarked below. All the descriptors described above can be computed for all modules using the phylogenetic_statistics function and exported as a csv file, where each row corresponds to a module.

Profylo also implements a go_enrichment function designed to facilitate gene set analysis of the modules. Given an annotation file for the proteome, it uses the goatool library to test GO term enrichment in each module, and outputs the first five terms in order of ascending Benjamini–Hochberg False Discovery Rate (FDR), along with the FDR value. The output of this function is also a csv file where each row corresponds to a module. Both of the above functions are designed to help users explore the results of Profylo.

#### Profile Visualization

Profylo provides several options to output the results of the module prediction. The first visual representation is a heatmap (profils_heatmap) of the presence-absence matrix for each module individually. To make them easier to interpret, profiles can be automatically ordered in taxonomic order (order_by_tree), allowing the user to easily identify the relevant pattern of shared losses. Additionally, clades of interest can be shown on top of the heatmap to provide context. This representation is designed to identify the major presence-absence trend explaining a module by providing a broad view of its constitutive profiles. Users can use the plot_modules function to automatically generate such heatmap figures as png files for all modules, for later visual investigation.

Alternatively, users may be interested in the specific nodes in a species tree where major shifts in presence-absence occur (i.e. tree nodes where genes are lost together). Since every gene evolutionary history is unique, providing this in a non-interactive plot is a challenge. Instead, Profylo outputs an annotated species tree in the extended Newick format using the tree_annotation function. For leaves (species), the annotation corresponds to the proportion of genes from a module present in the species. Similarly, ancestral nodes are colored with the average proportion value of all descendant species. Such trees can then be visualized using interactive tree visualization softwares, like phylo.io (Robinson et al. 2016), where the branches of the species tree with many absences can be outlined (Supplementary Fig. 1). This provides an interactive way to understand the evolutionary history associated with each module.

### Benchmarking

By offering many of the main methods for phylogenetic profile comparison, Profylo allows to easily benchmark the performances of different similarity or distance measures for a given task, as well as the impact of adjustable parameters on these performances. Here, we used the ability to identify genes sharing the same KEGG pathway (M. Kanehisa and Goto 2000) through the evolutionary signal as a benchmark for the seven implemented methods, their adjustable parameters and for Profylo’s parsimony score. This benchmark is similar to the one introduced in the work of Franceschini et al. (2016).

We used human phylogenetic profiles for different sets of species, in order to see how the species selection might affect the performances of individual methods. The datasets were derived from species available in the OrthoInspector (OI) database. The three species sets were: the 1472 eukaryotic species from the Eukaryota OI database (Eukaryotes), 600 species from the Transverse OI database covering the three domains of life, selected to represent the diversity of species and include high quality proteomes (Models), and the Eukaryotic subset of this last dataset with 258 species (EukModels).

We computed the evolution of precision against the number of true positives found, obtained by varying similarity thresholds for a positive prediction (see methods). Since all genes in a pathway do not necessarily co-evolve, we do not expect to obtain a high precision overall, however we assume that the best methods at capturing co-evolution signals will perform better, all else being equal, and maintain a high precision as recall increases and more true positives are found.

#### Global Benchmarking of Profile Comparison Methods

**Fig. 2:**
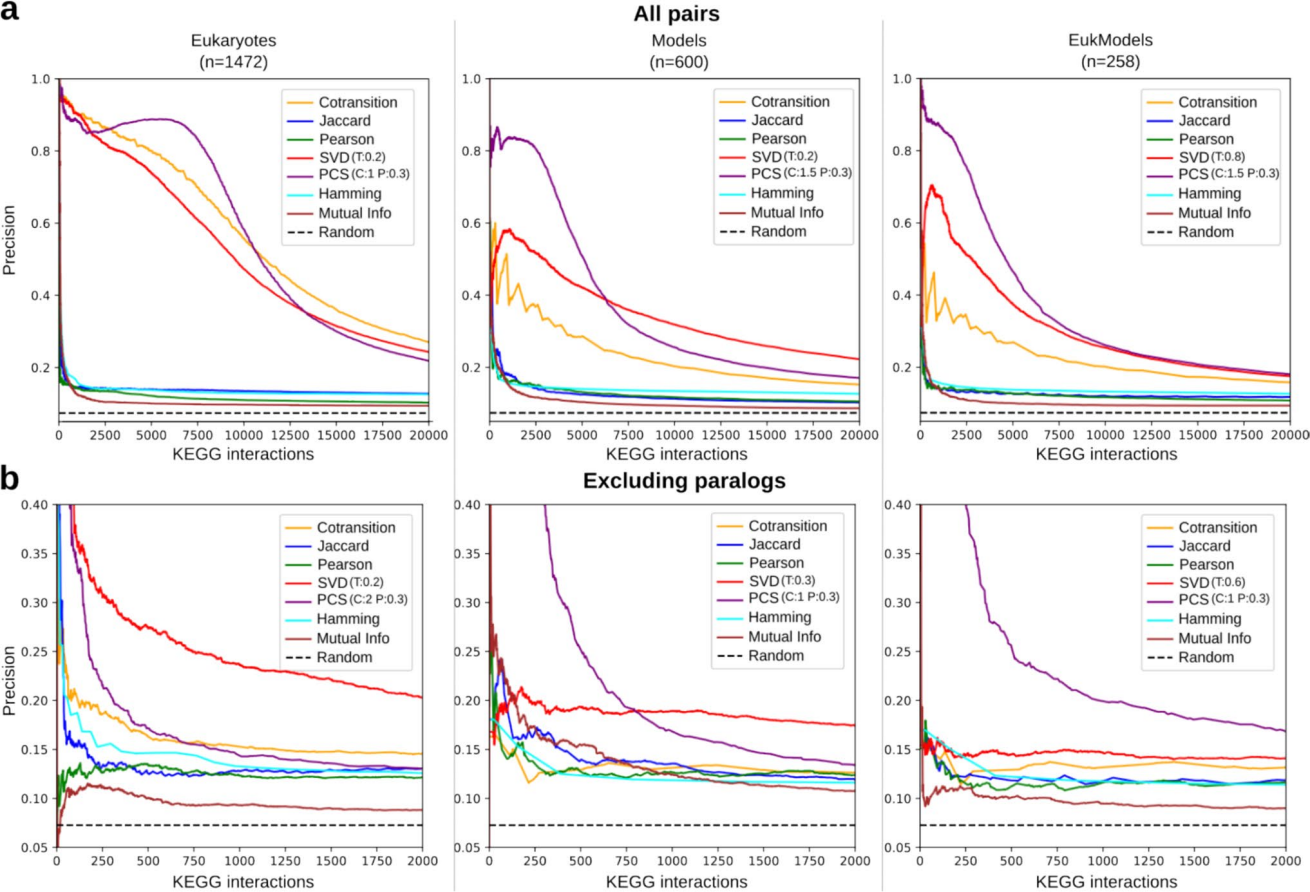
Benchmarking of phylogenetic profile similarity or distance metrics. a. Benchmark results including all gene pairs. b. Benchmark results when excluding all pairs between paralogous genes. The subplots show the number of positive KEGG interactions between human genes, as retrieved within the most similar profile on the x-axis, while the y-axis represents the precision within all pairs found with the same or higher similarity. Each subplot shows the results using a different species set for building the profile (Eukaryote: 1472 eukaryote species, Models: 600 model species, EukModel: 258 model eukaryote species). The methods which best capture functional association between genes are expected to retain high precision while identifying more positives. For SVD and PCS methods, the result for the best combination of parameters is shown. The legend indicates values for their parameters—T: truncation threshold in SVD-phy, C: confidence weight for PCS, P: penalty value for PCS)

We compared the performance of the 7 implemented methods using the benchmark described above and considering all pairs, including those between paralogous genes (see below for an analysis excluding them). The result of this analysis is shown in Fig. 2a, for the first 20,000 protein pairs. In order to compare the performance of the different methods with an objective criterion, we computed the area under the curve (AUC) for all methods (Supplementary Table 1). For these global results, we selected the parameter yielding the best results for the SVD-phy and PCS methods (see below). The performances of all methods depend on the species selected to build the profile, with notable differences between our three datasets and the best performances being attained on the Eukaryota dataset. However, in all three cases, the co-transition, PCS and SVD-phy methods are the highest performing methods with a wide margin over the other tested methods. In all cases, they maintain a precision of 0.2 or higher even when considering all 10,000 pairs, whereas other methods drop below this precision before the 1000 best ranking pairs. Among these three best ranking methods, PCS offers the most stable performances across all three datasets, maintaining the best AUC on all datasets.

When considering all pairs of proteins within a species, similarity of profiles is not necessarily attributable to co-evolution but rather to a shared evolutionary history due to paralogy. Indeed, close paralogs may have identical or highly similar profiles simply because they share the same evolutionary history prior to an ancestral duplication event. Since homologs, including paralogs, tend to share similar functions, it is necessary to control for this when assessing methods aimed at detecting co-evolution. Accordingly, we repeated the benchmarks as described above, but removed any pairs between genes detected as paralogous in the OrthoInspector database. The results are shown in Fig. 2b and the AUC in Supplementary Table 1.

When controlling for paralogy, the performance of all methods is worse, with a rapid decrease in precision as more pairs are considered. The gap in performance between the co-transition method and the methods based purely on binary profile comparison is smaller when excluding paralogs, but both SVD-phy and PCS (using the best performing parameters) tend to attain better results. Performances of individual methods differ depending on the datasets selected, with the PCS methods obtaining the best AUC on the two model databases (Models and EukModels) but not on the Eukaryotes one. SVD-Phy appears to perform in the opposite way, being the best method for the Eukaryotes dataset but having moderate performance on the Model ones. This may indicate that both methods are impacted by species number and data quality.

In terms of overall ranking, PCS consistently retains a higher precision than the other methods in the top few hundred true positives it identifies. This can be explained by the fact that the other scoring schemes systematically give the best ranking to identical profiles, which puts a ceiling on the precision they can reach. In contrast, PCS is not bound to an interval ([0;1] or [-1;1]) and its score increases with the number of shared evolutionary transitions, as a result the best ranking profiles are not necessarily identical but must share a high number of evolutionary transitions, and are most likely to truly co-evolve.

#### Effect of Parameter Choice

Two of the implemented methods, SVD-phy and PCS, use adjustable parameters in their calculations. In Profylo, these parameters are set by default to the values described in the respective original publication of each method, but the software allows users to change these parameters. For SVD-phy, the adjustable parameter is the truncation threshold, which corresponds to the portion of the SVD matrix that is retained before computing the euclidean distance between vectors. This parameter can be attributed values in the]0,1] interval (default = 0.3). For PCS, two parameters can be adjusted: the confidence weight parameter that adds more weight to matches or mismatches that are supported by more than two species (default = 1.5) and the penalty parameter that sets the weight of mismatches in transition between the considered vectors (default = 0.6).

We benchmarked the performance of different parameters and parameter combinations for these methods, using the same framework as above. Full results are shown in Supplementary Figs. 1–4. In SVD-phy, the effect of the truncation threshold change appears similar across all datasets including paralogous pairs: reducing this threshold leads to lower precision when considering only the highest ranked pairs, but to slower decrease in precision as more pairs are considered. Despite similar dynamics, the best performance is attained at 0.2 for the Eukaryota dataset for the Eukaryota and Model dataset but at 0.8 for the EukModel dataset.

On the benchmarks excluding paralogous pairs, the trend associated with the change of value is less clear. For the Eukaryotic and Models benchmark, the low thresholds of 0.2 and 0.3, respectively, give the best results as they retain the highest precision as more pairs are considered. In the EukModels benchmark, the optimal value of this parameter is 0.6.

For the PCS method, within the range of values we tested (1, 1.5, 2 for confidence weight; 0.3, 0.6, 0.9 for penalty weight), the penalty weight changes have the greatest impact on results. Specifically, high penalty weight leads to high precision for the highest ranking pairs in most benchmarks but a more rapidly decreasing precision as more pairs are considered. In all cases the best performances are obtained for a confidence weight of 1.5 and a penalty of 0.3. When paralogs are excluded, the relative difference between parameter values is less pronounced. As with paralogs, a penalty value of 0.3 consistently leads to better results, although the best confidence value depends on the benchmark, with better performance at a value of 1.5 for Eukaryotes and at 1 for the Model datasets.

#### Benchmarking of Parsimony Score

Profylo implements a measure of the number of events needed to explain a given profile under a Dollo framework parsimony—which we term parsimony score..This tool is designed as a way to prioritize modules with genes sharing eventful evolutionary history (with many gain or loss events), for which similar profile is indicative of plausible coevolution Although it is challenging to benchmark the usefulness of such a metric to help identify modules of functionally integrated genes, we used our KEGG pairs benchmarks to test whether such a score was useful to discriminate functionally associated pairs from non-functionally associated pairs.

First, for every pair in our datasets, excluding those between paralogs, we compared the distribution of the minimal parsimony score from genes in the pairs between positive and negative pairs (Supplementary Fig. 5). When all pairs are considered, we detect a significantly higher score for positive pairs relative to negative pairs (Mann–whitney one-sided test p-value = 0.00). However, this difference might be attributed to genes in the positive dataset being “older”—having appeared in a distant common ancestor—than ones in the negative set. Indeed, when only considering pairs of genes whose origin can be attributed to the last common ancestor (LCA) of all the considered species, no significant difference is detected (Mann–Whitney one-sided test p-value = 1.00).

Next, we considered whether differences in parsimony scores could be detected between positive and negative pairs, using any of the 7 similarity or distance metrics. We ranked all pairs descending from the LCA, excluding paralogous pairs, and classified them in deciles, from closest to farthest. Then, we compared the distribution of parsimony scores between positive and negative pairs. Despite the difference in distribution between deciles, we again found no evidence of parsimony scores being higher for positive pairs within each decile (Supplementary Fig. 6–8).

Next, we focused on the 2000 highest-ranked pairs for each method, for which relatively high precision values are attained as shown in the global benchmark (Supplementary Fig. 9–11). When doing this, we observed meaningful differences in parsimony scores between positive and negative pairs. Specifically, highest ranking pairs are more likely to have high parsimony scores than negative pairs for most methods except co-transition, PCS and SVD-phy. In those cases, both positive and negative pairs tend to have similarly high parsimony scores.

Thus, the parsimony score may be helpful in discriminating whether two genes are involved in the same pathway, but only when using metrics that are not themselves based on a model of species relatedness.

## Case Study

As an example case study, we used Profylo to extract and explore co-evolutionary modules within the human proteome. For this example, we chose the parameters based on performance on our benchmarking results. We computed the all-against-all matrix of PCS results with confidence parameter set to 1 and penalty set to 0.3 on human phylogenetic profiles extracted from OrthoInspector, with the EukModel species set and excluding paralogs. This combination of method and dataset was the one yielding the highest AUC on the KEGG benchmark. We used the Profylo make_modules function to extract profiles with the label_propagation method, setting the threshold to 7. We chose this value based on our benchmarking results, since this value corresponds to a precision of 0.31 and 331 positive pairs found, close to the inflection in the precision curve shown in Fig. 2b.

We characterized the profiles by performing Ontology (GO) term enrichment analysis for each module using the go_enrichment function in Profylo. We also used the phylogenetic_statistics of Profylo (explained above) to obtain simple descriptors of each module (size, common ancestor of the module, mean number of orthologs, parsimony score) and the plot_module function to obtain a graphical representation of each module. All files are available as Supplementary Tables or as part of the associated archive. Below is a summary of the results.

We obtained 389 distinct modules as the output of the clustering, covering a total of 5063 genes. The size of the modules are imbalanced with the biggest module covering a total of 1785 genes, but 344 modules having a size lower than 10. Among these clusters, 148 (38%) show functional enrichment for at least one GO term (with a Benjamini-Holchberg False Discovery Rate (FDR) lower than 0.05) signaling a significant overrepresentation of genes associated with the same biological process, molecular function or cellular component. This is dependent on the size of the clusters, with 66% of modules with more than 5 members having an enrichment compared to 27% of those with less than 5 members., The top 10 modules with the lowest FDR are shown in Table 2 along with their top enriched terms. The phylogenetic profiles of genes involved in these modules are available as Supplementary Figs. 12–21. All results of the enrichment analysis, including other significantly enriched GO terms, breakdown of results by GO categories and enrichments in genes involved in KEGG modules or pathways are available as Supplementary Table 2.

**Table 2:** Descriptors and Gene Ontology term enrichment of the first 10 modules, ranked by ascending False Discovery Rate

ID	Size	Parsimony score	Presence count	GO ID	Go Term	FDR
1	1785	14	69	GO:0005515	protein binding	0.0
2	754	30	113	GO:0005929	cilium	5.9e-181
16	19	21	227	GO:0009083	branched-chain amino acid catabolic process	3.4e-40
27	13	45	38	GO:0036128	CatSper complex	2.2e-37
20	17	118	80	GO:0098803	respiratory chain complex	1.4e-33
11	25	27	17	GO:0002250	adaptive immune response	2.1e-31
3	277	15	53	GO:0005515	protein binding	4.4e-28
5	45	19	30	GO:0019814	immunoglobulin complex	1.0e-26
19	18	14	232	GO:0008137	NADH dehydrogenase (ubiquinone) activity	8.3e-26
72	7	13	238	GO:1,901,663	quinone biosynthetic process	1.7e-21

**Fig. 3:**
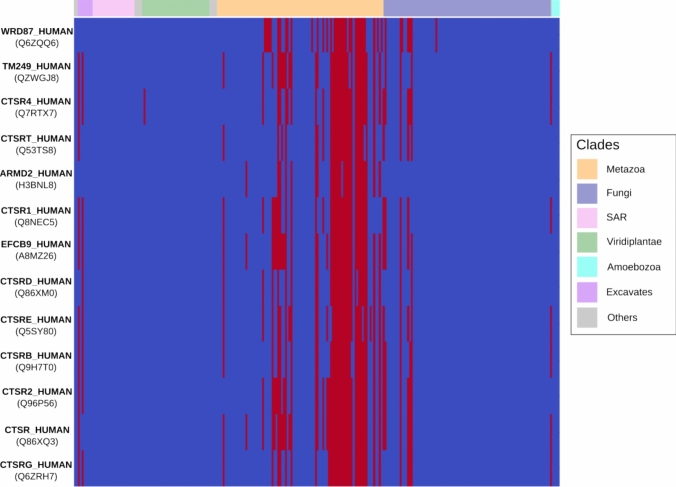
Example heatmap of the phylogenetic profiles for module 27 enriched in genes involved in the CatSper complex. Each row represents the profile of a gene (represented by its protein identifier and access), and each column to a species. The colored section on top corresponds to the taxonomic division of species

Two of the largest modules (1 and 3) have a significant enrichment in the protein binding GO term, which is one of the most basal Molecular Function terms and may mean these broad modules do not contain genes that are integrated in a single pathway. The second biggest module (2), containing 754 genes, is significantly enriched in the more informative cilium GO term and corresponds to a coevolutionary signal of genes involved in the eukaryotic cilium, which is known to be easily detected by phylogenetic profiling (see (Nevers et al. 2017)). The other modules all have a smaller number of members, and are associated with more specific GO terms. Remarkably, some of them contain all or most genes involved in known molecular complexes, such as the endosomal WASH complex (module 16) or the CatSper complex (module 27, shown in Fig. 3).

Users may be interested in exploring modules in more details through targeted analyses. The statistics provided by Profylo, as well as the visualization tools, can facilitate such analyses. For example, module 20 (17 genes, heatmap shown in Supplementary Fig. 13) shows a strong enrichment in the respiratory chain complex, and targeted analyses show this module includes 12 mitochondria-encoded genes and 3 nuclear-encoded mitochondrial genes. Analysis of the descriptors provided by Profylo shows that it is also the cluster with the highest parsimony score in the whole dataset. Visualization of the taxonomically sorted heatmap of this cluster highlights the “patchy” distribution of genes in this module—with rapidly alternating presences and absences, suggesting many gene losses. In this particular case, as the mitochondrial genome is present in most eukaryotic species, such a pattern might be an artifact of some UniProt proteomes used in OrthoInspector not including mitochondrial genomes rather than a biologically relevant pattern. The second highest parsimony score in the top module is observed for module 27, which is incidentally known to have experienced several losses—or gains through horizontal gene transfer—in eukaryotes (Cai and Clapham 2008). These examples highlight the fact that Profylo descriptors and visualization tools can provide leads for more in-depth investigations.

## Discussion

Here, we described Profylo, a Python library dedicated to phylogenetic profiling based on the standards set in the literature. It includes several algorithms to compare binary and continuous profiles, functionalities for isolating and identifying co-evolving groups of genes, and features designed to facilitate their analysis. We first used the opportunity provided by having several similarity measures implemented in the same package to benchmark their performance under different parameters. Then, we illustrated the analysis features by applying these tools to the human proteome.

Phylogenetic profiling analysis, despite its apparent simplicity, depends on a variety of parameters which impact the results in a significant way (Deutekom et al. 2022). The first parameter is the source of the profiles themselves. Many phylogenetic profiling studies use a similarity-based approach, aimed at distinguishing homologs, with or without including the similarity score within the profile. This approach has limitations, as it may falsely recognize genes as being present in some species where only a distant paralog can be found. Other studies, this one included, chose to use orthology inference methods at greater computational cost. Even in this setting however, two paralogs resulting from a recent duplication will share most of their orthologs and will therefore often have similar phylogenetic profiles, without this being the result of independent co-evolution.

In this study, we addressed this question by comparing the results obtained when taking into account or disregarding the similarities between pairs of genes detected as paralogs by OrthoInspector. When paralogs are included, many more positive pairs are detected. This is expected because paralogs may be involved in similar biological processes and share the same evolutionary history due to shared ancestry. The ability to identify genes involved in the same pathway when retaining paralogs is therefore better for all methods, with greater differences in performance between methods. However, if we focus solely on detecting coevolution between genes, the results obtained after excluding paralog pairs are more informative.

We did not create an edge between paralogs when designing our module detection analysis, meaning co-evolution has to exist between non-paralogous genes in a module for it to be detected. Modules can still include paralogous genes but only if non-paralogous genes share similarity with multiple paralogs. Other studies have excluded close homologs before, either by not considering similarity between genes passing a sequence similarity threshold (Franceschini et al. 2016) or considering profiles of orthologous groups (Dey et al. 2015; Moi and Dessimoz 2023).

Our benchmarking results also highlight the importance of species selection for phylogenetic profile analyses. Using the same gene set and the same functional similarity measures, but considering the presence-absence in a different set of species, we obtained remarkably different results for each similarity metric. Although it is difficult to pinpoint the reasons for these differences, multiple factors may impact the results. The Eukaryotes dataset contains 1472 species, and a sampling bias for certain clades (738 species are Fungi, as example). The Models dataset contains 600 species, selected for clade diversity balance and genome quality. The EukModel contains only the 258 species shared by the two other datasets. Considering the Eukaryotes and Models datasets, the differences can be attributed to different taxonomic focus. The differences between Eukaryotes and EukModel datasets, however, are still high.

Sampling bias is one of the factors, as some of the methods, in particular the Jaccard similarity, are sensitive to the proportion of shared orthologs and including many closely related species will amplify their signal. Data quality may be another factor, since any incomplete or error-ridden gene set will have an effect on profiles, specifically by increasing the number of absences for affected species. This can have a strong effect on transition-based similarity measures. The PCS method, specifically, obtains better benchmarking results on the curated EukModel dataset than on the full Eukaryota dataset, whereas the performance of other methods such as SVD-phy appear to increase with the number of species included. Thus, we recommend careful species set selection and curation before a phylogenetic profile analysis. As our identification of a module corresponding to the mitochondrial genes demonstrates, bias in the data can have a dramatic impact on the final analysis.

When designing this tool, our goal was to provide a universal package that implements a wide variety of the methods described in the literature for phylogenetic profiling. However, we chose to focus on a specific set of methods so they could be usable in a similar framework. Therefore, we did not integrate some state-of-the-art alternatives. HOGProf (Moi et al. 2020) is a fast phylogenetic profiling method based on Hierarchical Orthologous Groups (Altenhoff et al. 2013). Rather than comparing presence-absence profiles, it estimates similarity between genes by comparing vectors of evolutionary events shaping a gene family, where every vector is a branch of a species tree. This takes advantage of Hierarchical Orthologous Groups output by methods like OMA (Altenhoff et al. 2023). The need to first obtain HOGs, and not simply classical binary or continuous profiles to use this method meant it could not be easily implemented in Profylo’s framework. Moreover, HOGProf is already available as a maintained Python package so we chose not to implement it.

Another recent development in the field of phylogenetic profiling concerns clade aware profiling methods, as introduced by (Stupp et al. 2021). The purpose of such methods is to consider phylogenetic profiles of different clades to predict potential functional links between proteins, assuming some function may be acquired later, and co-evolution to be meaningful in only certain clades for this reason. We believe using clade-level information separately, rather than computing one single similarity score is promising. However, we did not implement it in Profylo as it went beyond the scope we were aiming for. The main challenge when introducing such methods is combining similarity metrics from different clades in a way that increases the number of functionally associated genes detected. The optimal combination depends on the dataset, and the clades considered. Indeed, (Stupp et al. 2021) used machine learning models trained on preexisting functional associations to best combine those profiles. Introducing the ability to use machine learning in Profylo is an avenue for future development but was too complex for this initial release.

In order to facilitate downstream analyses, Prophylo provides tools to obtain descriptions of modules, through various descriptors and visualizations of evolutionary history and functional overrepresentation. One of the descriptors we chose is what we call the “parsimony score”, which corresponds to the number of loss events needed to explain the consensus profile of a module in a Dollo parsimony framework. As we showed in our benchmark, this descriptor can be useful to detect genes that share a biological pathway when using classical methods such as the Jaccard or Hamming distances, but not when using methods based on transition vectors. Given that these methods are also the ones with the best performance, this descriptor may not always be useful to users. However, as shown in our example, it can occasionally help detect unusual patterns in the data (in our case, the unexpected pattern of mitochondrial genes). The tools for module description available in Profylo may help to explore the results and identify outstanding results, but more in-depth analysis and integration of biological information from other sources is still needed to derive biological insights.

Even the tools made available, such as the GO term analysis must be manipulated with care. Indeed, more than half the modules we obtained in our use case had no significant GO term enrichment. This absence of proof for functional integration is dependent on the size of the modules. Indeed, low numbers of genes present in a module decrease the statistical power of gene set enrichment analysis. Other factors that may explain this lack of proof for functional integration may be lack of relevant annotations of the genes (134 genes of the 5063 genes in the modules have no GO term whatsoever) and similarity in transition profile occurring by chance rather than coevolution. Even when enrichment is available the most significantly enriched GO terms for the biggest module in our use case is “protein binding”, this GO term is two levels from the root in the Molecular Function GO and concerns many human genes (near 14,000 according to UniProt). Such an enrichment may thus simply mean the module is rich in genes with an annotation, which is expected for a module with genes found in most metazoans (Supplementary Fig. 12). Obtaining significant but uninformative enrichment is a risk with large module such as the module 1 of our use case, which may have been pulled together due to sharing transitions commonly occurring in human’s phylogenetic profiling—gain in metazoa, loss or no found orthologs in flatworms and tunicata.

Overall, Profylo is designed to be a comprehensive toolkit for comparative genomics, providing access to many state-of-the-art phylogenetic profiling methods described in the literature through a single interface. It complements other toolboxes that aim to democratize the use of phylogenetic profiling (Tran et al. 2018; Moi et al. 2020). We hope it will be useful for studying gene repertoire evolution while exploiting the growing numbers of available genomes across the tree of life. As phylogenetic profiling methods continue to emerge (Moi and Dessimoz 2023), we expect to continue upgrading its possibilities with new methods, while accounting for user feedback.

## Supplementary Information

Below is the link to the electronic supplementary material.

Supplementary file1 (XLSX 6 KB)

## Data Availability

Profylo is available online on Github at https://github.com/MartinSchoenstein/Profylo. Input data and data generated during the course of this work are available on Zenodo at: 10.5281/zenodo.15224590. All orthology data used in this work are based on the 2023 release of OrthoInspector and are available on the database website at https://lbgi.fr/orthoinspector/.
